# Adolescent Takayasu's arteritis with hypertensive intracerebral hemorrhage: a case report and literature review

**DOI:** 10.3389/fped.2024.1432362

**Published:** 2024-08-16

**Authors:** Fang Zhang, Bingzi Dong, Libo Yang, Jiaxin Liu, Jinfeng Zhan, Yukun Cui, Hua Lin, Yangang Wang, Wenshan Lv

**Affiliations:** ^1^ Department of Endocrinology and Metabolism, Affiliated Hospital of Qingdao University, Qingdao, Shandong, China; ^2^ Department of Endocrinology, Tai'an Central Hospital Affiliated to Qingdao University, Tai'an, Shandong, China; ^3^ Department of Anesthesiology, Dandong First Hospital, Graduate School of Jinzhou Medical University, Dandong, Liaoning, China; ^4^ Department of Radiology, Affiliated Hospital of Qingdao University, Qingdao, Shandong, China

**Keywords:** Takayasu’s arteritis, renal artery stenosis, hypertension, cerebral hemorrhage, adolescent

## Abstract

Takayasu's arteritis is a primary systemic vasculitis that affects predominantly large vessels, affecting the aorta and its major branches. We report a case of adolescent female patient who initially experienced numbness and weakness in her limbs, subsequently developing severe hypertension. Physical examination revealed uneven blood pressure in the limbs and a murmur in the auscultation area of the abdominal aorta without decreased pulses. Auxiliary examinations revealed diffuse blood vessel stenosis, leading to the diagnosis of Takayasu's arteritis. One month later, the patient was diagnosed with multiple cerebral hemorrhages following sudden impairment of limb movement. Digital subtraction angiography did not reveal any evident vascular malformations or aneurysms. Following surgery and biologic intervention with tocilizumab, the patient's condition improved, with no new bleeding episodes and stable blood pressure control achieved. We also reviewed the literature that have been previously reported with hypertensive intracerebral hemorrhage complicated by Takayasu's arteritis. We recommend that Takayasu's arteritis be taken into account when considering the hypertension in young patients. Timely vascular imaging and standardized treatment are imperative for diagnosing and managing effectively.

## 1 Introduction

Takayasu's arteritis (TA), also known as pulseless disease, atypical coarctation of the aorta, or aortic arch syndrome, is a rare chronic non-specific granulomatous inflammation of unknown etiology, primarily involving the aortic arch and its main branches (1). This disease tends to affect young and middle-aged women, with most patients under 40 years old, and a male-female prevalence ratio of about 1:4–9 (2). Clinical manifestations vary depending on the location and severity of the disease. Early-stage symptoms may include fever, dizziness, and headache, while characteristics related to blood vessel stenosis or occlusion may manifest in the later stages. Renal artery involvement and hypertension are common clinical features, with cerebral hemorrhage being a serious complication that significantly impacts patient prognosis, often resulting in high mortality rates. In this study, we present a case of an adolescent female patient with TA complicated by hypertensive cerebral hemorrhage. We also summarize similar cases, aiming to provide insights for the clinical diagnosis and treatment of this disease subtype.

## 2 Case presentation

### 2.1 General information

A 15-year-old female presented to our hospital in August 2023 with a history of headache for one-month and involuntary movement of right limbs for one-day. She experienced a sudden onset of breathlessness accompanying headaches a month prior, with unequal blood pressure of 158/107 mmHg in the left upper limb, 162/113 mmHg in the right upper limb, 170/140 mmHg in the left lower limb, and 183/146 mmHg in the right lower limb. After ultrasound and aortic enhanced CT investigations, an active stage of TA was suspected and treated with prednisone 10 mg qd and cyclophosphamide 50 mg qod. Although breathlessness improved, blood pressure remained poorly controlled. One day before admission, she developed sudden difficulty moving her right limbs, accompanied by non-projectile vomiting.

Past medical history: Approximately 2 months ago, she received traditional Chinese medicine treatment for symptoms of general weakness, numbness, and loss of appetite. Otherwise, her medical history is unremarkable, with no reported family history of genetic disorders.

During the physical examination: Her blood pressure was measured at 179/113 mmHg in the left upper limb, 177/111 mmHg in the right upper limb, 193/142 mmHg in the left lower limb, and 200/140 mmHg in the right lower limb. Her heart rate was 200 beats/min, but she had no pulse weakness. A vascular murmur can be heard in the auscultation area of the abdominal aorta. She appeared drowsy but was able to utter monosyllables. Pupils on both sides were equal in size and round, approximately 3 mm in diameter, with present direct and indirect light reflexes. Muscle strength in the right limb was assessed at level 2, while the left side was at level 4. Right tendon reflex was negative, while the left was positive. Pap's sign was positive on the right and negative on the left. Neck stiffness was not observed, and there were no signs of meningeal irritation.

### 2.2 Laboratory and imaging test

Upon admission, a comprehensive examination revealed the following results: aldosterone levels were 811.3 pg/ml (standing position) (normal range: 31–351 pg/ml), renin levels were >500 pg/ml (standing position) (normal range: 2.8–28.5 pg/ml), and potassium levels were 2.41 mmol/L (normal range: 3.5–5.5 mmol/L). Additionally, the erythrocyte sedimentation rate was 40 mm/60 min (normal range: 0–20 mm/60 min), C-reactive protein was 11.44 mg/L (normal range: 0–5 mg/L) and IgA levels were elevated at 3.14 g/L (normal range: 0.81–2.32 g/L). No obvious abnormalities were observed in IgG, IgM, complement levels (C3 and C4), rheumatoid factor, antineutrophil cytoplasmic antibody, antinuclear antibody, renal function, or liver function tests.

**Figure 1:**
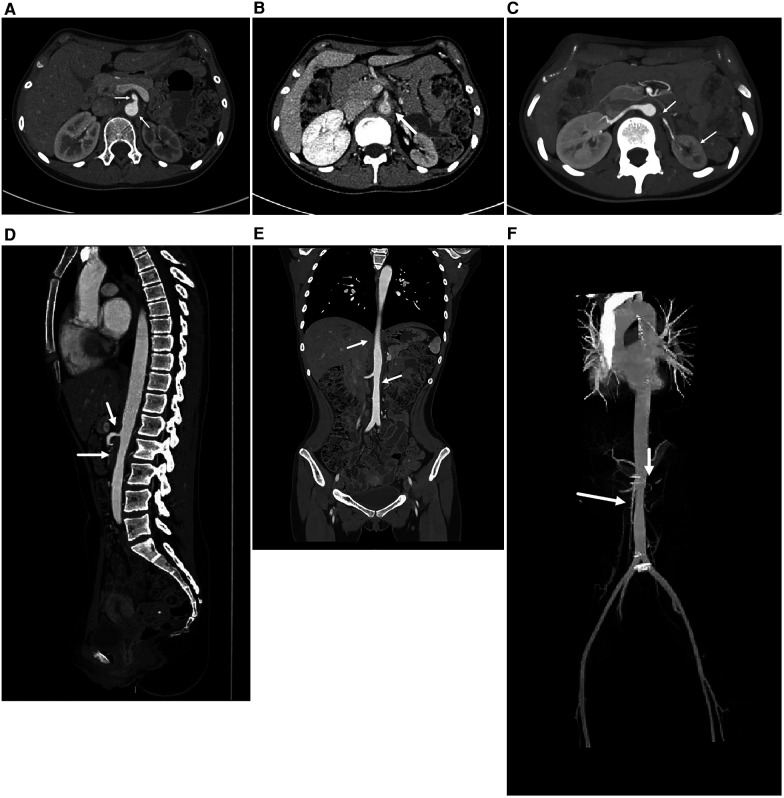
The enhanced CT image of the entire aorta. **(A,B)** The axial images of the abdominal aorta, A is the arterial phase and B is the venous phase. It can be seen that the walls of the abdominal aorta and superior mesenteric artery are thickened, leading to stenosis of the lumen. **(C)** The axial image of the renal artery. The lumen of the left renal artery is occluded and the left kidney is atrophied. **(D)** In the sagittal image, stenosis of the abdominal aorta and superior mesenteric artery lumen can be seen. **(E)** In the coronal image, partial stenosis can be seen at the abdominal aorta. **(F)** A more intuitive display of the stenosis of the abdominal aorta, with obvious renal artery level. (These images are provided by the Department of Radiology, Peking University First Hospital).

**Figure 2:**
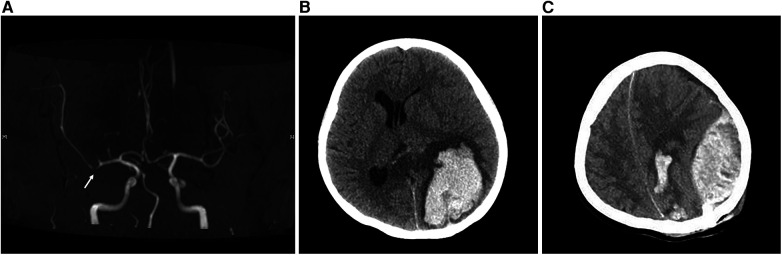
Brain MRA and brain CT images. **(A)** The MRA image of the brain shows visible stenosis of the M1 segment of the middle cerebral artery. **(B)** Preoperative CT image of the brain. Cluster-like high-density shadows in the left parieto-occipital lobe and the splenium of the corpus callosum, with unclear boundaries. The left lateral ventricle appears compressed and narrowed, and there is a rightward shift in the midbrain structure. **(C)** The brain CT image on the evening after surgery showed a left epidural hematoma.

**Figure 3:**
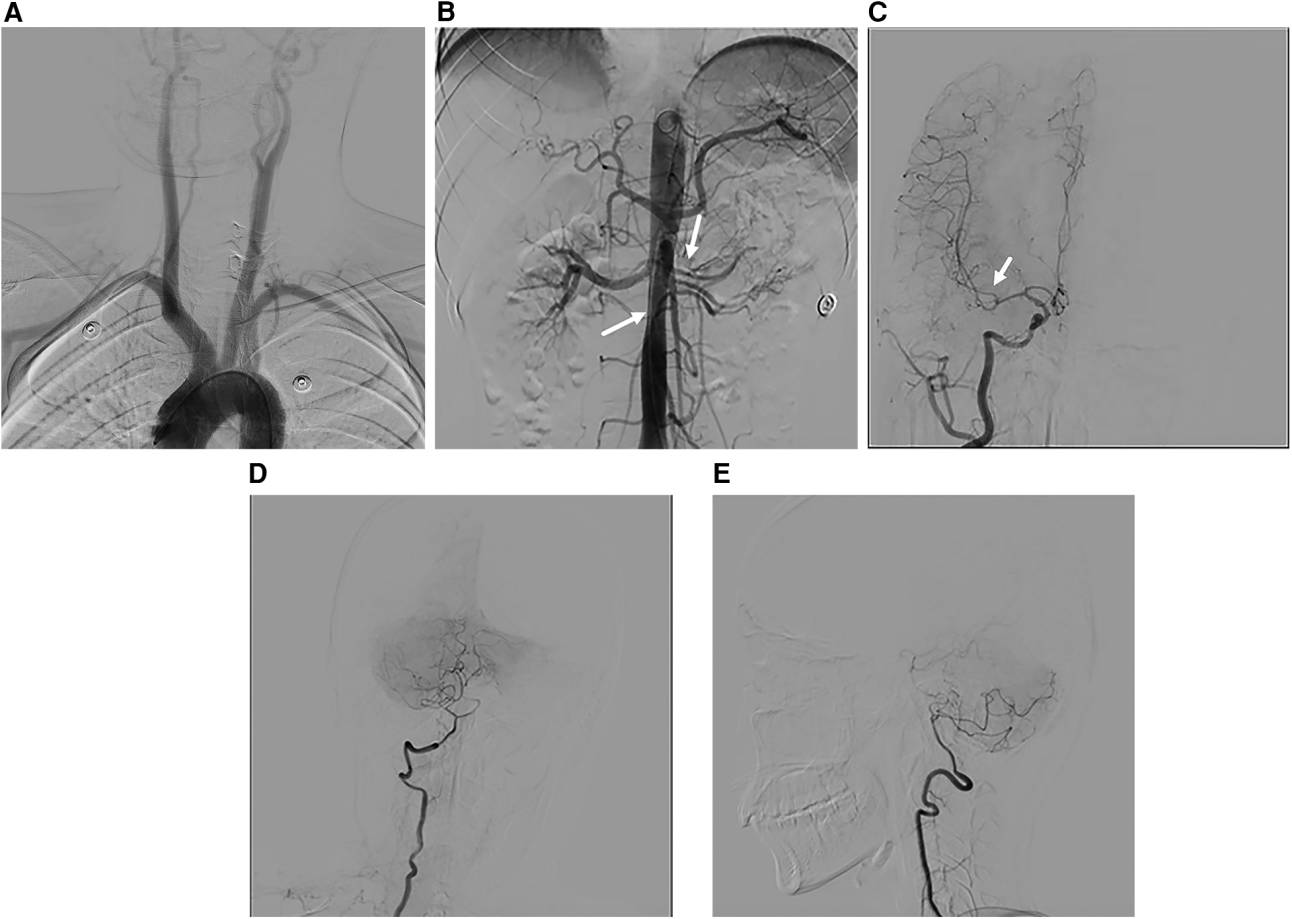
DSA images. **(A)** Image of the main branches of the aortic arch, with no obvious vascular stenosis. **(B)** Stenosis of the left renal artery and celiac trunk is evident. **(C)** The M1 segment of the right middle cerebral artery appears thinner than the M2 segment. **(D,E)** Anteroposterior and lateral view of the right vertebral artery, revealing a slender distal end of the intracranial segment.

Renal ultrasound revealed diffuse thickening of the wall of the middle and upper abdominal aorta, leading to stenosis of the initial segment of the left renal artery, consistent with Takayasu arteritis. Enhanced CT of the aorta showed mild stenosis of the abdominal aorta, occlusion of the beginning of the left renal artery and atrophy of the left kidney (Figure 1). Brain magnetic resonance angiography (MRA) showed multiple slender intracranial arteries (Figure 2A). Brain computed tomography (CT) revealed a high-density shadow with a maximum cross-sectional area of approximately 7.8 × 5.3 cm, consistent with brain parenchyma and subarachnoid hemorrhage (SAH) (Figure 2B). Digital subtraction angiography (DSA) was performed to assess the condition of the cranial blood vessels, revealing no obvious aneurysms or vascular malformations (Figure 3).

### 2.3 Diagnosis

The patient's aortic stenosis was observed on an aortic enhancement CT scan, along with unequal blood pressure in the extremities, vascular murmurs, hypertension, and elevated acute-phase reactants, leading to a definitive diagnosis of TA according to 2010 diagnostic criteria by EULAR/PRINTO/PRES (Table 1) (3). Because the patient's blood pressure was poorly controlled and no significant vascular malformations or aneurysms were evident on DSA, she was diagnosed with hypertensive cerebral hemorrhage.

**Table 1:** Final EULAR/PRINTO/PRES c-TA criteria (with glossary) and classification definition.

	Criterion	Glossary
Mandatory criterion	Angiographic abnormality	Angiography (conventional, CT, or MRI) of the aorta or its main branches and pulmonary arteries showing aneurysm/dilatation, narrowing, occlusion or thickened arterial wall not due to fibromuscular dysplasia, or similar causes; changes usually focal or segmental
One of the five criteria	Pulse deficit or claudication	Lost/decreased/unequal peripheral artery pulse(s) Claudication: focal muscle pain induced by physical activity
	Blood pressure (BP) discrepancy	Discrepancy of four limb systolic BP >10 mmHg difference in any limb
	Bruits	Audible murmurs or palpable thrills over large arteries
	Hypertension	Systolic/diastolic BP greater than 95th centile for heigh
	Acute phase reactant	Erythrocite sedimentation rate >20 mm per first hour or CRP any value above normal (according to the local laboratory)

### 2.4 Treatment and follow-up

Due to a significant hematoma causing pressure on the ipsilateral lateral ventricle, the patient underwent intracranial hematoma drilling and drainage. During the operation, dark black bloody fluid was observed flowing out. Three hours post-operation, the patient suddenly lost consciousness and failed to respond. A dilated right pupil compared to the left, along with a constricted left pupil, was noted. ECG monitoring indicated a blood pressure of 193/135 mmHg and a heart rate of 193 beats/min. A subsequent brain CT scan revealed an acute epidural hematoma (Figure 2C). Subsequently, evacuation of the epidural and intracranial hematoma, decompressive craniectomy, and intracranial electrode implantation were performed. The volume of hematoma removed during the operation was approximately 30 ml.

Following the procedure, sodium nitroprusside and esmolol were administered to lower blood pressure, accompanied by potassium and fluid supplementation, preventive anti-infection measures, nerve nutrition, and circulation improvement strategies. Additionally, after consultation with the Department of Rheumatology and Immunology, a 40 mg intravenous infusion of methylprednisolone was initiated to address the underlying disease. Due to the insufficient response to steroids and cyclophosphamide, the biological agent tocilizumab was introduced to manage the primary disease.

After six months of follow-up, the patient is currently taking carvedilol 12.5 mg twice daily, amlodipine 10 mg three times daily, and terazosin 1 mg once daily to manage blood pressure. To date, six doses of tocilizumab have been administered, and the hormone dose has been steadily reduced to 20 mg daily. Methotrexate 12.5 mg weekly is also being used for treatment. The blood pressure in the upper limbs is maintained at around 135/90 mmHg, and in the lower limbs at around 145/80mmHg. Muscle strength is at level 4 in the right limb and level 5 in the left limb. ESR and CRP were controlled within the normal range. However, some memory loss still remains. The patient's family is currently satisfied with the treatment results and is considering whether to undergo surgery for renal vascular stenosis.

## 3 Discussion

Cerebrovascular complications of TA typically stem from cerebral hypoperfusion caused by stenosis in the carotid or vertebral arteries (4). Clinically, the incidence of cerebral hemorrhage is much lower than that of cerebral ischemia, possibly due to collateral circulation that includes the thyroid cervical trunk, anterior spinal artery, the circle of Willis, and various unnamed blood vessels (5). An autopsy study of 10 patients in India indicated that cerebral hemorrhage is more common in individuals with renovascular hypertension (6). In this patient, DSA revealed no evident aneurysm or vascular malformation. Considering the patient's history of severe hypertension, it is presumed that the cerebral hemorrhage is associated with renovascular hypertension and is a complication of poorly controlled hypertension.

TA was initially described in 1908 (7). It is a disease with a global distribution, and its incidence varies among regions. The prevalence rate in the United States is 0.9 cases per million people, 0.4–1.5 cases per million people in Europe, and Japan has the highest prevalence, with about 40 cases per million people (8). However, the pathogenesis of TA remains unclear. Current understanding suggests that factors such as mycobacterium tuberculosis infection, autoimmune mechanisms, genetic predisposition, among others, all contribute to the development of TA (9, 10).

The clinical manifestations of TA are highly diverse. The acute phase is characterized by nonspecific symptoms, including fever, night sweats, anorexia, headache, and fatigue, among others. In the chronic phase, symptoms of end-organ ischemia may manifest, and complications such as aneurysm and dissection may occur (11). Although the patient in this case was found to have TA due to hypertension and hypokalemia, their history of fatigue and numbness is indicative of manifestations of the acute phase of TA. Hypertension is a common complication of TA and a primary reason for seeking medical attention in affected patients. The renal artery is frequently involved in TA-related hypertension, with clinical complications including malignant hypertension, ischemic nephropathy, cardiovascular and cerebrovascular diseases, renal atrophy, among others. TA has a 5-year survival rate less than 60% (12). Renal involvement is a significant predictor of adverse outcomes in TA, with factors such as renal insufficiency, bilateral renal artery involvement, and vascular stenosis >75% being associated with poorer prognosis (13). Therefore, early assessment of renal function in the disease course is crucial for reducing complications and improving prognosis.

Currently, only four cases of TA complicated by hypertensive cerebral hemorrhage have been reported, and their characteristics are summarized in Table 2 (14–17). A commonality among these cases is that they all involve female patients presenting with acute onset symptoms such as fatigue, numbness, and headache. Furthermore, all patients suffered from severe hypertension due to renal artery involvement, leading to cerebral hemorrhage resulting from poor blood pressure control.

**Table 2:** The comparison of five cases of hypertensive cerebral hemorrhage complicated by TA.

	Case 1	Case 2	Case 3	Case 4	Case 5
Age (years)	15	11	28	38	48
Sex	F	F	F	F	F
Duration of the disease	2 months	1day	14 years	10 years	7 years
Chief complaint	weakness, numbness, loss of appetite	hemiparesis, facial asymmetry	headache, abdominal pain	weakness, numbness, vomiting	headache, disappearance of arterial pulse
Hypertension	+	+	+	+	+
Aneurysm	–	–	–	–	AcomA
Affected arteries	RA, CA, MCA, VA	RA, AA	RA, AA, CIA, SMA	RA, CCA, SCA, ThAO, PA	RA, SCA, VA, AA
Location of bleeding	SAH, IPH, EDH	IPH	IPH	IPH	SAH (caused by aneurysm), IPH
Treatment	hematoma evacuation, medical treatment	medical treatment	medical treatment	medical treatment	hematoma evacuation, medical treatment
Result	under control	under control	under control	under control	under control
Ref.	Our patient	17	18	19	20

AcomA, anterior communicating artery aneurysm; RA, renal artery; CA, celiac axis; MCA, middle cerebral artery; VA, vertebral artery; AA, abdominal artery; CIA, common iliac artery; SMA, superior mesenteric artery; CCA, common carotid artery; SCA, subclavian artery; ThAO, thoracic aorta; PA, pulmonary artery; SAH, subarachnoid hemorrhage; IPH, intraparenchymal hemorrhage; EDH, extradural hemorrhage.

The special features of this case are noteworthy: Firstly, it has a childhood onset, with one patient being an 11-year-old child, while the remaining three are adults. Secondly, the disease course is remarkably short, progressing rapidly within only 2 months from onset to the discovery of cerebral hemorrhage. Except for case 2, cases 1, 3–5 all had a history of hypertension, with case 1 experiencing higher blood pressure for a shorter period. Thirdly, the condition is severe, complicated by SAH and epidural hemorrhages, in addition to parenchymal hemorrhages. Case 5 experienced a subarachnoid hemorrhage due to a ruptured aneurysm, while this case involved a non-aneurysmal subarachnoid hemorrhage. Only five cases of non-aneurysmal subarachnoid hemorrhage have been reported in patients with Takayasu arteritis (18, 19). The pathogenesis might be related to small vessel vasculitis and the susceptibility of neoplastic collateral vessels to rupture and hemorrhage, although further studies are warranted for confirmation (18). The occurrence of an epidural hematoma, typically associated with craniocerebral trauma, in this patient might be attributed to increased vessel wall fragility due to vascular inflammation and surgical injury during drilling and drainage. Finally, the treatment regimen for this case is complex, as it involves immunosuppressive therapy in addition to standard care, but fortunately, the patient responded well to tocilizumab, which is consistent with the efficacy of tocilizumab in the treatment of children with TA (20).

The onset of this patient's disease occurred in childhood. It has been reported that up to 30% of patients manifest symptoms during childhood. Clinical presentations and treatment strategies differ between childhood-onset TA and adult-onset TA. Children are more likely to exhibit constitutional symptoms such as fever and fatigue, and they tend to have renal artery involvement and hypertension. In contrast, adults are more prone to subclavian artery involvement, presenting with claudication, weakened pulses, unequal peripheral pulses and even absent pulses (21). Children typically experience shorter diagnostic delays but may require more frequent administration of immunosuppressants and biologics (22). Compared to adults, pediatric patients often exhibit more severe inflammation, extensive vascular involvement, and are at a higher risk of adverse complications (23).

Glucocorticoids are the first-line drugs for anti-inflammatory treatment in TA. In cases of refractory TA, treatment may be augmented with disease-modifying antirheumatic drugs (such as methotrexate, cyclophosphamide, etc.) or biological agents (such as tocilizumab, TNF-α inhibitors, etc.) (24). Surgical intervention should be considered when there are indications such as severe renal artery stenosis causing hypertension, claudication limiting daily activities, or cerebrovascular ischemia, preferably during the disease remission period. In recent years, research has highlighted the significance of signaling pathways in the pathogenesis of aortitis, suggesting that inhibitors targeting these pathways offer promising therapeutic prospects (25). In this case, although the patient received glucocorticoids therapy following the diagnosis of TA, the initial dose was inadequate, and antihypertensive medications were not promptly initiated to control blood pressure, consequently exacerbating disease progression. Hence, it is crucial for clinicians to commence standardized treatment early. Subsequently, surgical intervention to address renal artery stenosis becomes necessary once the condition is alleviated.

## 4 Conclusion

In summary, this article presents a case of a patient diagnosed with TA due to hypertension. However, insufficient understanding of the disease by clinicians resulted in a failure to conduct early vascular assessment and effectively control blood pressure, leading to disease progression and cerebral hemorrhage. Therefore, we recommend that for cases of unexplained hypertension, especially in young individuals, clinicians should conduct a comprehensive review of medical history, pay attention to early nonspecific symptoms, and perform a thorough physical examination to exclude TA as well as adrenal disease, renal disease or primary renovascular disease. Furthermore, personalized antihypertensive strategies should be implemented to mitigate the risk of hypertensive cerebral hemorrhage. Upon diagnosis of TA, early systemic vascular screening, appropriate corticosteroid therapy to induce remission, and revascularization during disease remission are essential.

## Data Availability

The raw data supporting the conclusions of this article will be made available by the authors, without undue reservation.
